# COVID-19: Mechanisms, risk factors, genetics, non-coding RNAs and neurologic impairments

**DOI:** 10.1016/j.ncrna.2023.02.007

**Published:** 2023-02-23

**Authors:** Irina Gilyazova, Yanina Timasheva, Alexandra Karunas, Anastasiya Kazantseva, Albert Sufianov, Andrey Mashkin, Gulnaz Korytina, Yaolou Wang, Ilgiz Gareev, Elza Khusnutdinova

**Affiliations:** a Institute of Biochemistry and Genetics, Ufa Federal Research Center of the Russian Academy of Sciences, 450054, Ufa, Russia; b Federal State Educational Institution of Higher Education, Ufa University of Science and Technology, 450076, Ufa, Russia; c Bashkir State Medical University, 450008, Ufa, Russia; d Рeoples’ Friendship University of Russia (RUDN University), 6 Miklukho-Maklaya Street, Moscow, 117198, Russia; e Department of Neurosurgery, Sechenov First Moscow State Medical University (Sechenov University), 119435, Moscow, Russia; f Harbin Medical University, 157 Baojian Rd, Nangang, Harbin, Heilongjiang, 150088, China

**Keywords:** COVID-19, Pathogenesis, Risk factors, Genetic, miRNA, lncRNA, Neurologic impairments

## Abstract

The novel coronavirus infection (COVID-19) causes a severe acute illness with the development of respiratory distress syndrome in some cases. COVID-19 is a global problem of mankind to this day. Among its most important aspects that require in-depth study are pathogenesis and molecular changes in severe forms of the disease. A lot of literature data is devoted to the pathogenetic mechanisms of COVID-19. Without dwelling in detail on some paths of pathogenesis discussed, we note that at present there are many factors of development and progression. Among them, this is the direct role of both viral non-coding RNAs (ncRNAs) and host ncRNAs. One such class of ncRNAs that has been extensively studied in COVID-19 is microRNAs (miRNAs) and long non-coding RNAs (lncRNAs). Moreover, Initially, it was believed that this COVID-19 was limited to damage to the respiratory system. It has now become clear that COVID-19 affects not only the liver and kidneys, but also the nervous system. In this review, we summarized the current knowledge of mechanisms, risk factors, genetics and neurologic impairments in COVID-19. In addition, we discuss and evaluate evidence demonstrating the involvement of miRNAs and lnRNAs in COVID-19 and use this information to propose hypotheses for future research directions.

## 1 Introduction

Currently, humanity is facing a global threat in the form of the COVID-19 pandemic caused by the new severe acute respiratory syndrome coronavirus 2 (severe acute respiratory syndrome coronavirus 2 - SARS-CoV-2). COVID-19 is characterized by a high level of morbidity and mortality among the elderly people and patients with comorbidities, especially diseases of the cardiovascular, respiratory and immune systems. Pathogenetically, COVID-19 is similar to two conditions characterized by the development of a cytokine storm and hyperactivation of the immune system, leading to target organ damage: the graft-versus-host reaction and macrophage activation syndrome [1]. Most patients have mild and moderate course of COVID-19, but in 14% of cases acute respiratory distress syndrome develops (ARDS) [2]. A large number of COVID-19 infected requires the involvement of significant resources of the healthcare system to provide adequate diagnostic assistance to all who needs computed tomography, the viral genome determination in the blood, including quantitative, serological studies followed by qualified medical care to prevent the development of complications [1].

## 2 Epidemiology and pathogenesis

One of the COVID-19 clinical features affecting the disease epidemiology is the non-symptomatic or paucisymptomatic course of the disease in most patients. An epidemiological study of 3,711 people who had quarantine period on board the Diamond Princess cruise ship after the SARS-CoV-2 detection in one of the passengers demonstrated that 17.9% of all infected people had non-symptomatic disease [3]. Another group of researchers found that the ratio of the number of people with clinical symptoms of the disease to the number of non-symptomatic individuals after a median incubation period of 14 days was 101:10000 [4]. According to the European Center for Disease Prevention and Control (ECDC), in the period from December 31, 2019 to December 18, 2022, 657.677.046 cases of COVID-19 were recorded in the world, including 6 671 707 deaths; thus, the mortality rate was 7.04%. In Europe, an average mortality rate of 202.4 per 1.000.000 populations is recorded, while there is a significant variation in the mortality rate in individual countries, from 2.6 in Slovakia to 523.6 in Belgium. Evaluation the severity of the disease course in different age groups by sex, such parameters as the frequency of hospitalization, the frequency of hospitalization in intensive care units and/or the need for respiratory support, total mortality and mortality among hospitalized patients was conducted. A significant increase in these indicators with age and predominance of men over women beginning in 30–40 years is noted [5]. According to the COVID-19 Surveillance Group, the median age of infected was 62 years, 85% of all deaths occurred between the ages of 70 and 89 years, while only 3.9% of patients had comorbid diseases. 15% of patients had one comorbid condition, 21.3% had two, 59.9% had three or more concomitant diseases (https://www.epicentro.iss.it). The most common comorbid conditions included previous myocardial infarction or stroke, atrial fibrillation, arterial hypertension (AH), diabetes, dementia, oncological diseases, chronic liver disease or kidney failure. Upon admission, 22.8% had no symptoms of the disease, 15.3% had a mild condition, 32.8% had a moderate condition, 15.2% had severe, 2.8% were in critical condition. According to international data, the most frequent clinical manifestations of COVID-19 included fever and dyspnea, cough, diarrhea, hemoptysis were less common, 96% with severe course of the disease developed ARDS, 33% had acute kidney failure. Superinfections were detected in 8.5% of critically ill patients, and the most frequent death cause was septic shock or macrophage activation syndrome [3,6]. At the earliest stages of the disease patients developed lymphopenia and neutrophilia. As the disease progresses, there was a further decrease in the number of lymphocytes, liver failure occurred with the hypoalbuminemia and a hyper inflammation state characterized by high levels of C-reactive protein, ferritin, D-dimer, lactate dehydrogenase, troponin and N-terminal fragment of brain natriuretic peptide precursor (NT-proBNP) [4,5]. It has been demonstrated that the pathogenesis of hyper inflammation is associated with an increase in the level of chemokines (C–C motif ligand 2 (CCL2), C–C motif ligand 3 (CCL3) and C-X-C motif chemokine ligand 10 (CXCL10)), cytokines (Interleukin-6 (IL-6), Interleukin-2 (IL-2), Interleukin-7 (IL-7), and Interleukin-10 (IL-10)) and granulocyte colony-stimulating factor (G-CSF), while the content of tumor necrosis factor alpha (TNF-α) is moderately elevated. Cytotoxic CD8^+^ and depleted T-lymphocytes, along with an altered balance of type 1 and type 2 T-helpers, reflect the development of severe immune system dysfunction [7]. The severity of the immune disorders is aggravated by the presence of a chronic low-intensity, flaccid inflammatory process characteristic of cardiometabolic diseases (coronary heart disease, arterial hypertension, obesity, type 2 diabetes) and respiratory tract pathology (chronic obstructive pulmonary disease). This leads to a high susceptibility to the virus and COVID-19 severe course in patients with chronic diseases of the cardiovascular and respiratory systems. Thus, it is fundamentally important to determine the pathophysiological mechanisms of the development of severe organ pathology under the influence of SARS-CoV-2.

## 3 The role of hereditary factors and comorbid diseases in the development of infectious diseases/COVID-19

There is a common misconception that hereditary factors and mechanisms play a minimal role in the development of infectious diseases. But humans have been in contact with microorganisms throughout their existence, and few other factors have had the same strong evolutionary influence on humanity. If a group of people encounters an infection for the first time, the contagiousness and mortality from it can be very high. An infectious disease development is a complex multicomponent process, where several genes control each step. In addition to monogenic inheritance of susceptibility to infections, there are oligogenic (e.g., susceptibility to leprosy, malaria, schistosomiasis) and polygenic inheritance patterns (susceptibility to tuberculosis). The predisposition to more frequent and less virulent infections is usually polygenic. In this case, the cumulative effect of a large number of genes is realized, and the contribution of each gene is relatively small. In general, the heritability of susceptibility to infectious diseases is a continuum from monogenic to multifactorial forms, and different individuals may have different genetic mechanisms controlling their susceptibility to the same infection. Natural recessive resistance to human immunodeficiency virus (HIV-1) results from genetic alterations in the chemokines genes CCR2, CCR3 and C–C chemokine receptor type 5 (CCR5), which serve as co-receptors to the CD4^+^ antigen of T-lymphocytes and are necessary for HIV-1 to enter the cell. In general, hereditary infection resistance due to pathogen receptor loss is a frequent (but far from the only) mechanism of innate resistance. The study of the molecular mechanisms of susceptibility and resistance to infectious diseases is extremely important for the development of new treatment approaches. COVID-19 is a new disease and there is limited information on risk factors for complications and adverse outcome. Current information and clinical experience suggest that the elderly people and patients with chronic diseases are at high risk for COVID-19 severe course (https://www.cdc.gov/coronavirus/2019-ncov/). According to recent studies, hypertension, diabetes mellitus, obesity, chronic obstructive pulmonary disease, cardiovascular disease (CVD) are independent factors in the development of severe complications in patients with COVID-19 [8]. Comorbid diseases are those diseases that are present simultaneously in one person. To date, significant evidence has accumulated pointing to a non-random combination of cardiovascular diseases, obesity, type 2 diabetes mellitus (T2DM) and chronic obstructive pulmonary disease (COPD). Several interrelated processes play a key role in the pathogenesis of all the above diseases: chronic systemic inflammation, oxidative stress, endothelial dysfunction. Any concomitant pathology contributes significantly to the overall severity of COVID-19 complications. According to a meta-analysis conducted by Zhao et al., patients with COPD and a significant smoking history have a high risk of developing COVID-19 severe form [9].

## 4 The role of angiotensin converting enzyme (ACE2) and CD147 receptor in the pathogenesis of COVID-19

It has been demonstrated that SARS-CoV-2 can use angiotensin-converting enzyme 2 (ACE2) as a receptor for viral entry into cells because ACE2 has an affinity for the S-glycoproteins of several coronaviruses, including SARS-CoV and SARS-CoV-2 [10] viruses. Much information has been accumulated on the role of ACE2 as a central regulator of blood pressure in the renin-angiotensin system (RAS), which regulates blood pressure, water-electrolyte metabolism, and systemic vascular resistance. Angiotensin II (Ang II) is the main effector of RAS, which has a powerful vasoconstrictor effect and stimulates aldosterone secretion by the adrenal glands. ACE2 is a membrane exopeptidase catalyzed the Ang II conversion which has vasoconstrictor, pro-inflammatory and fibrogenic effects which exhibits vasodilatory, anti-inflammatory and anti-fibrogenic properties [1,7,11,12]. In addition, ACE2 promotes the conversion of angiotensin I into angiotensin II [[1], [2], [3], [4], [5], [6], [7], [8], [9]], which has protective effects against cardiac and vascular remodeling and prevents the development of pulmonary hypertension by reducing apoptosis and inflammatory responses [13,14]. ACE2 is also capable to affect a number of other substrates. For example, bradykinin, which has a pro-inflammatory effect and Apelin, which is expressed on the surface of endothelial cells and realizes the hypotensive effect by inducing nitric oxide production. Also, the endogenous neuropeptide neurotensin, which has a vasodilatory effect by modulating dopamine signaling and the opioid receptor agonist dynorphin, the so-called “hunger hormone” ghrelin, which is produced in the GI tract and acts on several brain structures, including those that increase appetite [15,16]. ACE2 is expressed in the lungs and several other tissues, including the brain, kidneys, gastrointestinal tract, and adipose tissue. ACE2 expression was also found in the hypothalamus, pituitary gland, and adrenal gland, where the virus concentration was higher than in any other tissues besides the lungs [17]. In ACE2 transgenic mice, the olfactory bulb was the main target of SARS, leading to rapid transneural spread of infection [18,19]. This is consistent with data on frequent olfactory and taste disorders in patients with COVID-19 (85.6% and 88.0%, respectively) [20] It is reported ACE2 is expressed in a number of olfactory epithelial cells including supporting epithelial cells, basal epithelial cells, and Bowman gland cells [21]. It has been demonstrated that decreased ACE2 expression is associated with heart and kidney damage in AH, and ACE2 activation in the brain has neuroprotective effects in ischemic stroke [[22], [23], [24]]. SARS-CoV-2 infection can lead to toxic over accumulation of Ang II and bradykinin caused ARDS, pulmonary edema, and myocarditis by suppressing ACE2. ACE 2 presented on the surface of many cells of the respiratory system, including stem cells, has been shown to be involved in the development of pulmonary fibrosis [25]. SARS-CoV-2 can infect people of all ages, but the most severe disease forms develop in patients over 55 years with complex comorbid chronic diseases, such as coronary heart disease (CHD) and COPD. It was shown ACE2 expression in epithelial cells of the lower respiratory tract was significantly increased in patients with COPD and smokers [26,27]. SARS-CoV-2 binding to ACE2 suggests that long-term uncontrolled hyperglycemia, rather than a history of diabetes mellitus, may be important in the COVID-19 disease pathogenesis [28]. In addition, it should be noted that the ACE2 gene is located on the X chromosome, which may partially explain the predominance of males over females among COVID-19 patients [29,30]. In addition, differences in ACE2 gene methylation levels depending on gender were found CpG islands hypomethylation of the ACE2 gene was detected in women [31]. Tissue-specific methylation levels analysis also demonstrated the methylation level of the ACE2 gene was the lowest in lung epithelial cells compared to other tissues, and the methylation level of one of the CpG islands (cg08559914) located near the ACE2 gene transcription start site was significantly associated with biological age in respiratory tract epithelial cells [31]. It was found the increased an individual susceptibility to the SARS-CoV-2 virus may be the result of the combined effect of therapy and features of ACE2 gene polymorphisms (Table 1).

**Table 1:** Genetic variants associated with an increased infection risk of acute respiratory coronavirus infection.

ACE2 (rs200180615, C > T, c.2002G > A, c.1669G > A, p.Glu668Lys, p.Glu557Lys, T = 0.00053)	ACE2 (rs140473595, С>T, c.1501G > A, p.Ala501Thr, T = 0.000265)
ACE2 (rs2285666, C > T, g.8790G > A, T = 0.350199)	ACE2 (rs4646127, A > G, g.15597330A > G, A = 0.190728)
AHSG (rs4917, T > C, c.743T > C, с.746T > C, c.740T > C, p.Met248Thr, p.Met247Thr, p.Met249Thr, T = 0.264577)	ALOXE3 (rs147149459, G > A, c.1889C > T, c.2285C > T, c.1886C > T, p.Pro630Leu, p.Pro762Leu, p.Pro629Leu, A = 0.000399)
ERAP2 (rs150892504, C > G,T, c.2251C > G,T, c.2116C > G,T, c.2182C > G,T, p.Arg751Gly,p.Arg751Cys, p.Arg706Gly, p.Arg706Cys,p.Arg728Gly, p.Arg728Cys,T = 0.002596)	ANPEP (rs141945020, G > A,C,T, c.389C > T, c.389C > G, c.389C > A, p.Thr130Ile, p.Thr130Ser, p.Thr130Asn, T = 0.00004–229E coronavirus receptor)
BRF2 (rs138763430, C > T, c.25G > A, p.Asp9Asn, T = 0.001597)	ALOXE3 (rs151256885, C > G,T, g.8109899C > G, T = 0.020367)
CCL2 (rs1024611, A > G, g.34252769A > G, G-2518A, G = 0.363618, GG genotype)	CD14 (rs2569190, A > G, с-159CC, g.140633331A > G, A = 0.46885, CC genotype)
CYP4F3A (rs3794987, A > G, g.15640081A > G, G = 0.360823)	FGL2 (rs2075761, C > A,T, c.158G > A, c.158G > T, p.Gly53Val, p.Gly53Glu, T = 0.151757)
HLA-B*4601 (OR = 2.08, P = 0.04—disease severity)	HLA-B*5401 (OR = 5.44, P = 0.02—disease severity)
ICAM3 (rs2304237, T > C, c.428A > G, c.443T > C, C = 0.179513, TT, CT, CC)	ILA (rs1800587, с.–889T, TC, OR = 10.2, P = 0.031—increased viral load)
IL-4 (rs2070874, С>T, c.–33C > T, T = 0.401158)	IL-18 (rs1946518, T > G, c.–607, T = 0.407947, с.607T—increased viral load, TT, GT)
MBL (codon 54, A/B, B allele)	Mx1 (rs2071430, G > T, c.–88G > T, T = 0.238219, GT vs. GG—high risk)
RANTES (rs2107538, C > G, c.–28, T = 0.307708, G allele, GG genotype)	RelB (rs2288918, +23962T, TT, OR = 7.2, P = 0.034)

**Abbreviations:** ACE2, Angiotensin converting enzyme 2; AHSG, Alpha 2-HS Glycoprotein; ERAP2, Endoplasmic Reticulum Associated Aminopeptidase 2; BRF2, B-related factor 2; CCL2, C–C motif ligand 2; HLA, Human leukocyte antigen; ICAM3, Intercellular adhesion molecule 3; IL-4, Interleukin-4; MBL, Mannose-binding lectin; RANTES, Regulated upon Activation, Normal T Cell Expressed and Presumably Secreted; ALOXE3, Arachidonate lipoxygenase 3; ANPEP, Alanyl aminopeptidase, membrane; CD14, Cluster of differentiation 14; FGL2, Fibrinogen like 2; ILA, ILITHYIA; IL-18, Interleukin 18; Mx1, MX Dynamin like GTPase 1; RelB, RelB proto-oncogene.

SARS-CoV-2 has been found to penetrate the body by binding to the CD147 receptor [32]. The CD147 surface receptor (also known as Basigin; extracellular matrix metalloproteinase inducer (EMMPRIN) plays an important role in the host protection against both foreign antigens and its own antigens. The receptor is a major factor in the regulation of matrix metalloproteinase (MMP) expression.

CD147 is a pathophysiologically important multiligand immunoglobulin superfamily receptor expressed in various tissues and cell types, including leukocytes, endothelial cells, and platelets. CD147 was previously identified as a red blood cell receptor for binding to the malariae plasmodium parasite (Plasmodium falciparum) caused malaria in human. Particular interest of SARS-CoV-2 penetration pathway is due to the possibility of creating drugs targeting CD147, i.e. its potential druggability [33]. The use of humanized antibodies to CD147 has been demonstrated to reduce the virus penetration into host cells [33]. CD147 can increase MMP expression levels in bronchial asthma, COPD, and diabetic complications. CD147 and MMP expression levels are often elevated in inflammatory processes and tumors, reflecting the progression and metastasis of the tumor process. CD147 has previously been shown to be involved in the development of CHD [34]. Recent studies have shown that patients with severe asthma and COPD have high levels of matrix metalloproteinase 9 (MMP-9) in sputum, and influenza A virus infection increases CD147 expression in lung cells in bronchial asthma patients [32]. CD147 expression is also induced by high glucose levels, which contributes to an increase in the expression of matrix metalloproteases matrix metalloproteinase 1 (MMP-1), MMP-9. The key pathogenetic mechanisms in COPD and IHD are imbalance in the proteolysis-antiproteolysis system, increased activity and hyperexpression of matrix metalloproteases, caused by chronic systemic inflammation. Therefore, inhibiting CD147 may have a positive effect on the prevention of complications related to inflammation, including severe acute respiratory syndrome, which is the complication of COVID-19. CD147 is a second receptor for SARS-CoV-2 also presented on many cell types in the lung and is actively expressed in pneumocytes and type II macrophages at the edges of fibrotic zones [35].

## 5 Risk factors for severe COVID-19

AH is an important mortality risk factor globally, and its importance is further highlighted in the context of the new COVID-19 coronavirus infection. Patients with a severe course of COVID-19 usually have a history of AH [10]. The systemic chronic inflammation characteristic of AH, COPD, and CVD is also a pathogenetic touch point predisposing to the development of severe complications in COVID-19. Rapid deterioration in COVID-19 patients with is associated with a pro-inflammatory cytokine storm. COVID-19 patients have increased systemic levels of cytokines such as IL-2, IL-6, and IL-7, granulocyte colony-stimulating factor, and chemokines (CXCL10, CCL2, and TNF-α) [10]. Previously, our own studies and the published works by other authors have shown that the same factors play a key role in the pathogenesis of COPD, hypertension, CHD, and T2DM [[36], [37], [38], [39], [40], [41], [42], [43], [44], [45], [46], [47], [48], [49], [50], [51], [52]]. Accelerated aging of the immune system by age-associated diseases (AH, COPD, T2DM, and CHD) may part explain why the diseases in a patient's history are potentially associated with a more severe course of COVID-19. Therefore, major studies analyzing the relationship between age-associated disease and COVID-19 are urgently needed.

There is strong evidence elevated levels of oxidative stress and the transition of pulmonary inflammation into the systemic bloodstream play an important role in the pathophysiology of COPD and its comorbid conditions [53]. Oxidative stress is an important mechanism in the development of COPD, CVD, and metabolic disorders. The levels of oxidative stress biomarkers in patients with COPD are increased in exhaled air condensate, serum, and systemic blood flow. Exacerbation increases the level of oxidative stress. Oxidant levels increase not only as a result of smoking and particle inhalation, but also during the activation of inflammatory cells [macrophages and neutrophils] [[54], [55], [56]]. Increased levels of oxidative stress may be associated with decreased levels of endogenous antioxidants in patients with COPD due to lower levels of the transcription factor nuclear factor erythroid 2-related factor 2 (NRF2). It belongs to the redox-sensitive signaling system Kelch-like ECH-associated protein 1 (KEAP1)/NRF2, which is involved in the regulation of transcription of most antioxidant genes. The main hypothesis of systemic inflammation in COPD studied by many researchers is the migration of inflammatory mediators from the lungs into the systemic bloodstream [[57], [58], [59]]. One mechanism can be related to the high permeability of pulmonary vessels in COPD and leads to the release of pro-inflammatory factors into the blood in COPD patients, smokers and former smokers contributed to the transfer of local inflammation into the systemic bloodstream. The mechanism is associated with peripheral vascular endothelial dysfunction [[60], [61], [62]]. Smoking is a trigger mechanism for the development of endothelial dysfunction leading to systemic inflammation. Hypoxia and hyperinflation are common pathophysiological changes in COPD and may be associated with systemic inflammation. Serum concentrations of IL-6, TNF-α, monocyte chemoattractant protein-1 (MCP-1), and macrophage inflammatory protein-1β (MIP-1β) increase in hypoxia. In hyperinflation, systemic levels of TNF-α, IL-8, IL-6, and Interleukin-1beta (IL-1b) increase. Another mechanism is the release of a specific pulmonary protein into the systemic bloodstream, the surfactant D (SP-D). Obesity has been found to be a major risk factor for severe complications and death in COVID-19 [63]. During the H1N1 pandemic in 2009, obesity was classified as a risk factor for hospitalization due to the need for mechanical ventilation and high mortality [64]. Currently, elevated glucose levels are known to be associated with a high risk of complications and death in infections such as SARS and H1N1 influenza [65,66]. Adverse outcome of infections in obese patients is related to difficulties in intubation and the excessive body weight leads to increase pressure on the diaphragm and difficulty breathing. It is known obesity is associated with the development of chronic meta-inflammation even in the absence of an infectious agent, which has adverse effects on the immune system [67]. Due to the growth the COVID-19 pandemic, studies of molecular mechanisms of the relationship between obesity and the development of life-threatening complications are required. The obesity degree, abdominal obesity, hypertension, lipid spectrum indicators, carbohydrate metabolism disorder and markers of systemic inflammation (e.g., CRP and fibrinogen) influence on the clinical course of acute respiratory viral infections, the formation of bacterial complications in the bronchopulmonary system and features of the period of recuperation in patients with metabolic syndrome. Obese individuals are known to be more susceptible to infection due to complications development and high death probability. It can be explained lower T-lymphocytes response on the background of obesity. Obesity affects the efficiency of the immune system and magnitude of the immune response. Overweight provokes chronic inflammation, progressed with age, makes difficult to breathe and increases the need for oxygen. It was found body weight loss in patients with metabolic syndrome and obesity reduces the level of systemic inflammation markers, normalizes lipid and carbohydrate metabolism, reduces the frequency of recurrent acute respiratory infections (ARIs) and has a positive effect on the severity of the course of acute respiratory viral infections. According to Petrilli et al., patients with body mass index (BMI) over 40 kg/m2 and heart failure develop serious COVID-19 complications [68]. Moreover, obesity correlates with the risk of severe pneumonia even more strongly than cardiovascular or pulmonary disease [68]. Widespread overweight, over a third of the world's population as well as T2DM makes matters worse [69]. The current pandemic conditions are extremely dangerous for obese individuals, so it seems important to investigate the molecular mechanisms of obesity contributed to the more severe course of COVID-19. Obesity is accompanied by the development of T2DM, hypercholesterolemia, AH, etc. These complications also contribute to the more severe course of COVID-19 [70,71].

## 6 Genetic markers of severe COVID-19

Drucker et al. identified changes in several gene expressions in various metabolic pathways of pulmonary epithelial cells, such as increase leptin and insulin secretion, with decrease adiponectin activity [72]. The authors identified the several altered metabolic pathways, including IL-10 signaling pathway responsible for the acute inflammatory response to microbial proteins, negative regulation of apoptosis, acute phase response, TNF-signaling, other cellular pathways related to homeostasis, cell migration, vascular morphogenesis, activation of matrix metalloproteinases [72]. Also alterations of genes expression regulated glucose metabolism and adipogenesis (adipocyte markers: Leptin, Leptin receptor (LEPR) and adiponectin (ADIPOQ)) are detected [73]. SARS-CoV-2 causes changes in the host lipid profile. Existing obesity is associated with an abnormal lipid profile and SARS-CoV-2 can lead to increased susceptibility to infection. When the virus enters the cell, it leads to a deterioration of the lipid profile and causes further hyperinflammation. Leptin is a peptide hormone of adipose tissue regulated energy metabolism, has an anorectic action and participates in the regulation of glucose homeostasis and regulation of the immune system. Leptin stimulates fatty acid oxidation and can lead to lipotoxicity by reducing lipid accumulation in adipose tissue. The authors showed that among the pathways whose expression was elevated, the most pronounced changes were characteristic of genes regulating glucose metabolism and regulation of adipogenesis [72]. Most obese patients were found to have leptin resistance to both the leptin receptor and its cytokine signal transduction suppressor-3 (SOCS-3) inhibitor. Previous studies have demonstrated that obesity is associated with leptin resistance and increased leptin levels with a concomitant increase in SOCS-3, involved in signal transduction inhibition of leptin and several cytokines [74]. The increase in the level of adipogenesis is caused by an increase in the proliferation and differentiation of adipocytes. Increased proliferation and differentiation of adipocytes raises the level of adipogenesis. Peroxisome proliferator-activated receptor (PPAR) genes, activated peroxisome proliferators, and other RNA expression factors associated with adipogenesis, among others, are responsible for this process [64]. Leptin receptor is expressed in the liver, pancreas, oral mucosa. The Insufficiency leads to the development of leptin resistance, leading to obesity. One of these genes is the LEPR gene, which encodes leptin receptors. Leptin, a hormone produced by adipocytes and a number of other tissues, such as the gastric mucosa, acts as a signaling molecule involved in the regulation of body weight. When leptin reaches the brain, it acts on hypothalamic receptors, leading to a decrease in appetite, stimulating energy intake and weight loss. Leptin performs its function by binding to leptin receptors, which belong to the cytokine receptor family. Primarily, these receptors are located in the hypothalamus; they are also found in tissues and cells that regulate glucose homeostasis, such as pancreatic beta cells. In this case, leptin receptors influence leptin-mediated inhibition of insulin secretion. Thus, leptin, as a signaling factor produced by adipocytes, plays an important role in metabolism, controlling blood glucose uptake by tissues, fat oxidation and reduction of food intake. We identified the association of the LEPR polymorphism Gln223Arg with the development of obesity [75]. Previous studies have also demonstrated association of the LEPR polymorphism with glucose metabolism [76] and insulin resistance [77]. Adiponectin has attracted the attention of scientists because it has antidiabetic and anti-atherogenic effects, as well as its anti-proliferative action [78]. It has a powerful insulin-sensitizing effect by binding to adiponectin receptor 1 (ADIPOR1) and adiponectin receptor 2 (ADIPOR2) receptors and leads to the activation of adenosine monophosphate activated protein kinase (AMPK) and thereby promotes fatty acid oxidation and glucose assimilation in muscles, as well as suppression of gluconeogenesis in the liver [79]. Plasma adiponectin level is decreased in patients with visceral obesity and insulin resistance. Hypoadiponectinemia is a predictor of T2DM, hypertension, and atherosclerosis progression [80]. A decrease in body mass index leads to an increase in adiponectin in the blood [81]. The ADIPOQ gene, which is localized on chromosome 3, encodes the amino acid sequence of adiponectin (3q27). There is considerable allelic diversity of this gene in various world populations. Nucleotide sequence changes in the gene considered as potentially functionally significant are localized in the promoter region (−11426A > G rs16861194, -11391G > A rs17300539, -11377C > G rs267729), in exon 3 (T415C, Tyr111His, rs17366743), and in intron 2 (+276G > T, rs1501299). They are associated with the mRNA level of the ADIPOQ gene and adiponectin secretion [82]. Peroxisome proliferator-activated receptor α (PPARα) is expressed mainly in tissues with high levels of fatty acid catabolism, including liver, brain, brown fat, white adipose tissue. The PPARα regulates genes responsible for fatty acid metabolism, is responsible for glucose metabolism. PPARα is involved in the inflammatory response in vascular atherosclerosis. The peroxisome proliferator-activated receptor β (PPARβ)/peroxisome proliferator-activated receptor δ (PPARδ) is known as a nuclear hormone receptor. It plays an important role in fatty acid oxidation processes. The PPARβ/δ is an important mediator of insulin sensitivity [83] and is directly related to the process of obesity and myocardial hypertrophy through inhibition of nuclear factor kappa B (NF-kB). In vivo and in vitro studies have shown the critical role of the receptor in the regulation of adipocyte differentiation and lipid accumulation in adipose tissue in maintenance of the viability and normal function of differentiated adipocytes. The peroxisome proliferator-activated receptor γ (PPARγ) can promote the conversion of macrophages into foam cells by increasing the uptake of oxidized low-density lipoproteins (LDL). This contributes to atherogenesis. The peroxisome proliferator-activated receptor gamma 2 (PPARG2) gene encodes the PPARγ nuclear receptor. Various studies have demonstrated the association of the genetic variant of this gene with the T2DM development [84]. PPARγ receptor is a molecular target for thiazolidinedione compounds related to sugar-lowering drugs. The gene is expressed in adipose tissue, in a protein encoded by the PPARG2 gene. The rs1801282 single nucleotide polymorphism, characterized by the replacement of cytosine for guanine at codon 12, leads to exchange of proline to alanine (Pro12Ala) at the protein. Approximately 15% of Caucasians have alanine at position 12 of the PPARG2 protein product. This causes increased transcriptional activity, increased insulin sensitivity, and protection of the risk of T2DM development [85]. Many negative findings followed the first studies. In familial studies, using the transmission disequilibrium test (TDT), excessive transmission of the Pro allele to future generations with T2DM was observed [86]. Thus, COVID-19infection has a significant impact on the pathways involved in lipid metabolism. Decreased regulation of fat metabolism is a characteristic of obesity, one of the risk factors for COVID-19. The increased susceptibility may be caused by an increase in ACE2 expression in the lungs, regulated by sterol regulatory element-binding protein 1 (SREBP-1). The increased susceptibility may be caused by an increase in ACE2 expression in the lungs, regulated by SREBP1. Based on the results of several other studies conducted, we can suggest possible predicted biomarkers to the development of severe COVID-19 (Table 2) (see Table 3).

**Table 2:** Genetic factors of severe COVID-19.

HLA-B*46:01/A25:01/C01:02	Hypoexpression of the CXCR6 gene
Polymorphism of the CCR9 gene (rs11385942-GA)	X-chromosomal TLR7 gene (g.12905756_12905759del and g.12906010G > T)
rs150892504 mutation of the ERAP2 gene	rs12252 allele of IFITM3 gene
ACE-2 wild-type or naïve expression	ACE-2 allele P.Arg514Gly
ACE-2 alleles p.Arg708Trp, p.Arg710Cys p.Arg710His, p.Arg716Cys	Overexpression of the SLC6A20 gene
Polymorphism of the IFNAR2 gene (rs2236757)	TMPRSS2 p.Val160Met allele
Polymorphism of the ABO gene (rs657152)	Polymorphism of the TMEM189-UBE2V1 gene (rs6020289-A)
Polymorphism of the IFITM3 gene rs12252-C/C	Polymorphism of the PIEZO gene rs7184427, rs6500495 and rs7404939
Polymorphism of the OAS1, OAS2 and OAS3 genes	Polymorphism of the DPP9 gene (rs2109069)
Polymorphism of the TYK2 gene (rs2109069)	

**Abbreviations:** HLA, Human leukocyte antigen; ACE-2, Angiotensin converting enzyme 2; IFNAR2, Interferon alpha/beta receptor 2; IFITM3, Interferon-induced transmembrane protein 3; OAS, 2′-5′-Oligoadenylate Synthetase; TYK2, Non-receptor tyrosine-protein kinase 2; CXCR6, C-X-C chemokine receptor type 6; TLR7, Toll-like receptor 7; SLC6A20, Solute carrier family 6 member 20; TMEM189-UBE2V1, Plasmanylethanolamine desaturase; PIEZO, Piezo type mechanosensitive ion channel component 1; DPP9, Dipeptidyl peptidase 9.

**Table 3:** Summary of published studies on the role of microRNAs (miRNAs) in COVID-19, with relevant literature highlighted.

MiRNA	Study design	Object	Gene-targets	Biological function	Reference
miR-98	Bioinformatics In vitro	HMVEC-L HUVEC	TMPRSS2	Modulates TMPRSS2 expression in the endothelial cells	[115]
miR-1207-5p	Bioinformatics In vitro	Human alveolar and bronchial epithelial cells	CSF1	Enhances inflammatory responses in COVID-19 patients, and promoting EMT, which can contribute to pulmonary fibrosis, a possible sequela of COVID-19.	[116]
miR-28-3p	In vitro	293 T cells	Disintegrin and ADAM17	Exerts its function on both cell viability and cell apoptosis (Inhibit miR-28-3p expression inhibited cell viability and promoted cell apoptosis during S-protein treatment)	[117]
miR-31, miR-29, miR-126, and miR-17	Bioinformatics In vitro	Human serum samples	ZMYM5, COL5A3, and CAMSAP1	These miRNAs have been down-regulated and the levels of their mRNA targets have been enhanced with the increase of disease grade	[118]
miR-148a	In vitro	HEK-293T and CHME3	USP33, IRF9, TNFα, NF-κB, and IFN-β	Activate human microglia	[119]
let7b‐5p	In vitro	Naso‐oropharyngeal swabs and ATCC	ACE2 and DPP4	Participates in the mechanisms of acquiring the virulence of the virus and perform as a therapeutic target for COVID‐19	[120]
miR-125b-5p and miR-155-5p	In vitro	Human serum samples	CDH5, STAT3, and TRIM32	Participates in the mechanisms of acquiring the virulence of the virus. Could be useful a novel potential biomarkers to predict the time nodes of the acute, turn-negative, and recovery stages of virus	[121]
miR-32-5p, miR-98-3p, miR-423-3p, and miR-1246	Bioinformatics In vitro	Human serum samples	ACE2 and RAB14	Could be taken as potential biomarkers of COVID-19 progression as well as candidates for future therapeutic approaches. Relates to viral infection, inflammatory response, and coagulation-related processes	[122]
miR-29a-3p	RNA-Seq analysis In vitro	PBMCs	THBS1 and IL17RA	Potentially valuable candidate for predicting clinical manifestations of COVID-19, in the context of severity	[123]
miR-155	In vitro	Whole blood samples	SOCS1	Promote severity of COVID-19 and facilitates immune inflammation	[124]

**Abbreviations:** HMVEC-L, Lung microvascular cells; HUVEC, Human umbilical vein endothelial cells; CHME3, Human microglial cell line; ATCC, HeLa cell line; PBMCs, Peripheral blood mononuclear cells; TMPRSS2, Transmembrane protease serine 2; CSF1, Colony stimulating factor 1; ADAM17, Metalloproteinase 17; ZMYM5, Zinc finger MYM-type containing 5; COL5A3, Collagen type V alpha 3 chain; CAMSAP1, Calmodulin-regulated spectrin-associated protein 1; USP33, Ubiquitin specific peptidase 33; IRF9, Interferon regulatory factor 9; TNF-α, Tumor necrosis factor-alpha; NF-κB, Nuclear factor kappa-light-chain-enhancer of activated B cells; IFN-β, Interferon-β; ACE2, Angiotensin-converting enzyme 2; DPP4, Dipeptidyl peptidase-4; CDH5, Cadherin 5; STAT3, Signal transducer and activator of transcription 3; TRIM32, Tripartite motif-containing protein 32; RAB14, Ras-related protein; THBS1, Thrombospondin 1; IL17RA, Interleukin 17 receptor A; SOCS1, Suppressor of cytokine signaling 1.

The pathogenesis of COVID-19 has been demonstrated to be linked to the development of coagulopathy. It differs from the disseminated intravascular coagulation syndrome characterized by septic conditions. For COVID-19, almost normal levels of prothrombin, fibrinogen and platelets are seen, despite a significant increase in dimmer D [87,88]. At the same time, the main pathogenetic mechanism of coagulation disorders in COVID-19 is the development of pulmonary intravascular coagulopathy (PIC) [89]. Autopsies of dying COVID-19 patients showed signs of right ventricular insufficiency resulting from pulmonary hypertension, fibrin organization in the alveoli, protein exudate and infiltration by monocyte-macrophage cells. It indicates the early development of coagulopathy and explains the effectiveness of traditional therapies in such patients, which leads to high mortality [90]. SARS-CoV2 binding to ACE2 receptors on the surface of type II pneumocytes and vessel endothelial cells results in cell lysis and immediate endothelial activation. This is accompanied by the development of a procoagulantive condition and affects the accumulation of fibrin deposits in pulmonary microcapillaries [91]. Fibrin deposition first triggers a mechanism of increased plasminogen activity, then decompensation occurs, manifested by higher levels of D-dimer. When the thrombosis process involves larger vessels, secondary pulmonary hypertension, cardiac failure, and severe hypoxia occur. As a result of the impaired venous flow, the left ventricular stroke volume decreases, which leads to the development of AH. In patients with numerous comorbidities [coronary heart disease, arterial hypertension, obesity, after kidney transplantation] who died from COVID-19, the pathological anatomical examination revealed signs of lymphocytic endotheliitis in lungs, heart, kidneys, liver and vessels, as well as hepatocyte necrosis [92]. During their lifetime, COVID-19 patients developed acute mesenteric ischemia, requiring resection of the small intestine, and histological examination revealed evidence of endotheliitis and apoptotic cells in the intestinal vessels [92]. The endothelial cells express ACE2, which is a receptor for SARS-CoV-2 [93]. COVID-19 is more severe in older adults and is asymptomatic or mild in children and young adults. However, in a number of cases, COVID-19 diagnosed in children and adolescents (2–15 years of age) has manifested as a multisystem inflammatory syndrome resembling Kawasaki disease or toxic shock. Patients developed rash, abdominal pain, vomiting, and diarrhea. Less than half of patients had a respiratory condition. Children with a genetic predisposition to COVID-19 are presumed to have a severe course of the disease with predominantly gastrointestinal lesions, but it is not yet known which features of the genotype lead to the development of this form of the disease [94]. Currently, there is no doubt about the importance of the genetic component in the development of serious complications of COVID-19, especially in patients with comorbid pathology. There is a need for research to identify relationships at the level of pathogenic molecular mechanisms and for an integrated systematic approach to solve the problem. It will combine assessment of a set of clinical and instruments, biochemical parameters, analysis of the level of expression of a network of interrelated key genes, environmental exposures, and the contribution of genetic factors. The project involves identification of molecular markers and evaluation of their role in the development of severe complications in COVID-19 in patients with comorbid pathology. A comprehensive strategy based on a combination of genetic associative studies of known candidate genes for COPD, CVD, T2DM with analysis of the key genes expression profile involved in insulin signaling, immune and inflammatory response, oxidative stress in circulating blood cells will be used. Analysis of a complex of protein biomarkers implicated in inflammation and immune response will also be carried out. For molecular markers selected by our team, our own studies and results of other researchers have previously shown an association with the development of age associated pathologies including cardiovascular disease, chronic obstructive pulmonary disease, obesity, and T2DM. In addition, several studies have demonstrated that selected genes are involved in cellular aging.

## 7 Non-coding RNAs in COVID-19

Non-coding RNAs (ncRNAs) refers to ribonucleic acid (RNA) molecules that do not code for a protein. They cover a huge number of RNA classes and perform a wide range of biological functions, such as regulation of gene expression, protection of the genome from exogenous DNA, and control of DNA synthesis. One such class of ncRNAs that has been extensively studied are microRNAs (miRNAs) and long non-coding RNAs (lncRNAs) [95]. MiRNAs are endogenous and non-coding single-stranded RNAs of 18–22 nucleotides that inhibit gene expression by promoting degradation of their messenger RNA (mRNA) targets or inhibition of translation. It has been proven that miRNAs play an essential role in various biological processes, including the cell cycle, apoptosis, cell proliferation and differentiation, regulating the expression of about one third of all human genes [96]. LnRNAs are a group of ncRNAs longer than 200 nucleotides. Because of the nucleotide chain lengths of lncRNAs have the unique ability to take on many complex secondary and tertiary structures, allowing them to perform specific functions such as catalysis, metabolism, and cell differentiation. LncRNAs cannot code for protein, but can modulate gene expression at epigenetic (e.g., DNA methylation, histone modification), transcriptional (e.g., recruitment of transcription factors), and post-transcriptional (e.g., regulation of miRNA and mRNA stability) levels [97]. An increasing number of studies have demonstrated deregulation or aberrant expression of miRNAs and lncRNAs in a variety of diseases, including infection disease [98]. Mane studies have shown that miRNAs and lncRNAs play a role a direct role in the virus life cycle [[99], [100], [101]]. Many viruses, including COVID-19, encode and express their own miRNAs and lncRNAs in host cells. Some of these miRNAs and lncRNAs modulate the activity of the immune system and facilitate the escape of the virus from its protective action, as well as maintain the latent infection. A number of viral miRNAs and lncRNAs are complementary to the host mRNA and, conversely, many miRNAs and lncRNAs of the host are complementary to the virus genes. The interaction of the virus and the host at the level of these regulatory elements is important in the pathogenesis of COVID-19 infection. About 200 miRNAs and about a few dozen human lncRNAs have potential antiviral activity [[102], [103], [104]]. Complementarity to these ncRNAs was found in most viral genomes, which may indicate the presence of ncRNA-mediated antiviral protection in humans. In this chapter will focus on the roles of miRNAs and lncRNAs in COVID-19 infection and antiviral responses and underlying regulatory mechanisms.

### 7.1 MiRNAs in COVID-19

Currently, scientists are increasingly paying attention to the study of miRNAs molecules that are able to regulate gene expression. Thus, miRNAs are able to play a critical role in a wide range of biological processes, and have previously been shown to be important effectors in complex “host-pathogen” interaction networks. To date, there is no complete understanding of the molecular mechanisms triggered by miRNAs in cells infected with the COVID-19 infection. Previous studies have shown conflicting results: on the one hand, intracellular miRNAs can inhibit viral replication, and on the other hand, in some viruses, including COVID-19, mechanisms have emerged that allow avoiding the inhibitory action of the host miRNAs. There are a number of studies that have studied the mechanism of miRNA-induced repression of the immune response, in reaction with the COVID-19 protein [105,106].

MiRNAs play an important role in the regulation of the viral cell cycle (miR-152-3p, miR-219a-1-3p, and miR-644a), are responsible for the induction of innate immunity (miR-148a and miR-590), and are involved in the development of B- and T-lymphocytes (miR-151a-5p, miR-223, miR-29, miR-15a-5p, miR-199a-3p, miR-103a, and miR-155) [[107], [108], [109]]. It is known that miR-2392, miR-4476, miR-223, and miR-200c can suppress viral gene expression and inhibit COVID-19 virus replication in vitro [110,111].

COVID-19 latency in resting target cells with ACE2 receptors located on cells of the respiratory tract, heart, esophagus, kidney, bladder, ileum, nervous system, is the most significant obstacle to virus clearance in patients receiving antiviral therapy [17]. As already mentioned, a number of cellular miRNAs inhibit the production of COVID-19 [109]. Specific inhibitors of these miRNAs enhance the production of COVID-19 proteins and the production of new virions in target cells isolated from COVID-19-infected patients receiving antiviral therapy. Thus, host cell miRNAs play an important role in inhibiting replication and establishing latency of COVID-19, and restoring dysregulation of miRNA expression may be a promising approach to control the virus and eliminate it from latent reservoirs [105,106].

**Fig. 1:**
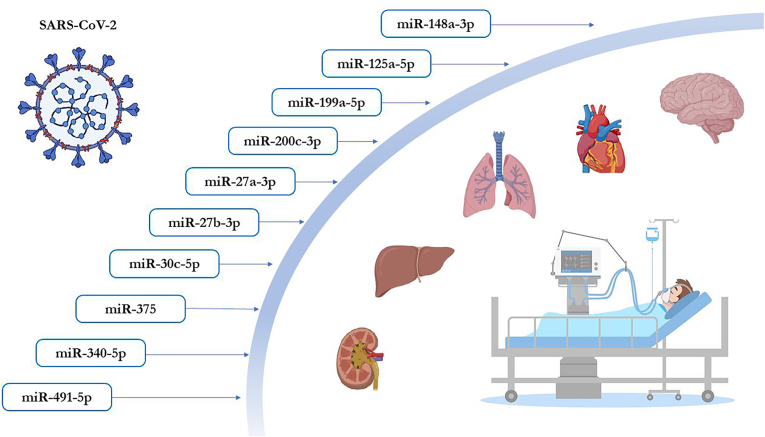
MicroRNAs (miRNAs) and severe COVID-19 infection. MiRNAs can act as autocrine, paracrine, and endocrine cellular regulators. MiR-199a-5p, miR-27a-3p, miR-27b-3p, and miR-491-5p correlate with increased plasma cytokine tumor necrosis factor-alpha (TNF-α), interleukin-1 beta (IL-1β) and interleukin-6 (IL-6) storms. MiR-146a, miR −146b and interleukin-8 (IL-8) result in acute respiratory distress syndrome and chronic obstructive pulmonary disease (COPD) observed in severe COVID-19. MiR-148a-3p downregulates the expression of the target ubiquitin-specific protease 33 (USP33) gene and subsequent levels of interferon regulatory factor 9 (IRF9) and upregulates the expression of a major pro-inflammatory gene profile of TNF-α, nuclear factor kappa B (NF-κB), and interferon-β (IFN-β), which induces inflammation in the lungs. MiR-200c-3p targets the expression of the angiotensin-converting enzyme 2 (ACE2) gene, which codes for ACE2, involved in the regulation of blood pressure, thereby negatively affecting the overall health of the cardiovascular system. The immune response is also affected by miR-30c-5p and miR-340-5p, which regulate viral replication during a viral infection.

With COVID-19 infection, there is a violation of the expression miRNAs, however, they still play a significant role in counteracting viral infection (Fig. 1). Although monocytes and macrophages have the necessary receptors for COVID-19 entry into the cell, monocytes are quite resistant to COVID-19 infection in vitro. In contrast, tissue macrophages differentiated from monocytes in vitro have a high susceptibility to infection with certain strains of COVID-19. The infection rate of monocytes is quite low and they rarely serve as a reservoir for COVID-19. This feature of monocytes is due to the high content of antiviral miRNAs (e.g., miR-223), which is not observed in macrophages originating from them [[112], [113], [114]]. Suppression of the expression of antiviral miRNAs in monocytes facilitates their infection with the virus, and an increase in the expression of anti- COVID-19 miRNAs in macrophages leads to suppression of virus replication. These data provide a molecular explanation for the resistance of monocytes to COVID-19 infection and support the notion that innate immunity mediated by intracellular miRNAs may play a significant role in protecting cells of the immune system from COVID-19 infection. A brief summary of the major miRNAs and their regulatory effects involving signal pathways of COVID-19 mechanism is shown in Table 1 [[115], [116], [117], [118], [119], [120], [121], [122], [123], [124]].

### 7.2 LncRNAs in COVID-19

**Fig. 2:**
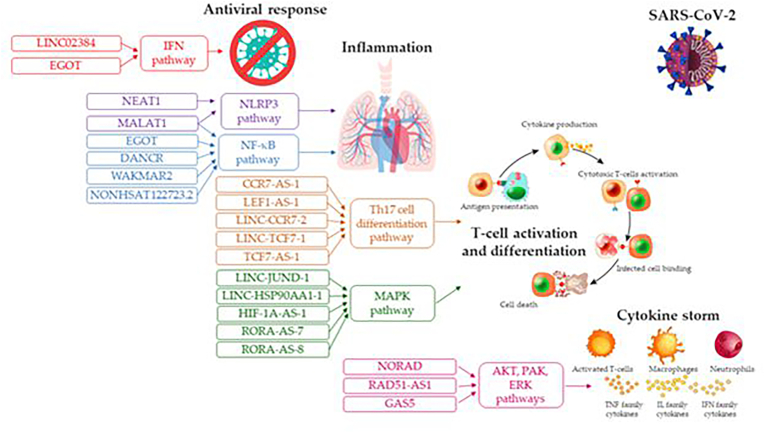
The long non-coding RNAs (lncRNAs) and signaling pathways affecting COVID-19 infection response. LncRNAs are new regulatory elements that can mediate interactions between host and pathogen during COVID-19 infection. Entering human organism, a virus activates pathogen recognition receptors and triggers innate immune response, upregulating the expression of type I interferons (IFNs) producing genes. Antiviral response to COVID-19 can also be regulated by lncRNAs through the IFN-related pathway. Some lncRNAs can promote the release of inflammatory cytokines triggering the cytokine storm often viewed as a hallmark of COVID-19 infection. Adaptive immune response to COVID-19 can also be regulated by lncRNAs by influencing T-cell activation and differentiation via Th17 cell differentiation pathway and mitogen-activated protein kinase (MAPK) pathway. The nucleotide-binding oligomerization domain (NOD)-like receptor pyrin domain containing 3 (NLRP3) and nuclear factor kappa B (NF-κB) are key signaling pathways in the development of the excessive inflammatory response and cytokine storm that are associated with COVID-19 severity and mortality, and are significantly affected by lncRNAs. Due to their involvement in crucial mechanisms of the COVID-19 infection, lncRNAs are promising candidates as prospective diagnostic biomarkers and therapeutic targets in COVID-19 treatment.

The results of many studies demonstrate that lncRNAs are involved in various biological regulatory processes such as inflammation, immune system disorders, and play an important role in the pathogenesis of various diseases, including viral infections [125]. In patients with COVID-19, a number of lncRNAs play an important regulatory role in the development of the virus [126,127] (Fig. 2). For instance, metastasis-associated lung adenocarcinoma transcript 1 (MALAT1) and nuclear paraspeckle assembly transcript 1 (NEAT1) have been shown to be strongly associated with immune responses and possibly involved in the progression of the inflammatory process in COVID-19 [126]. In addition, MALAT1 inhibited deep vein thrombosis (DVT) by inactivating the proliferation and migration of endothelial progenitor cells (EPCs) and was involved in thrombus dissolution through regulation of the Wnt/b-catenin signaling pathway [127]. In other words, lncRNAs become key regulators of the intense inflammatory response and thrombosis in COVID-19 patients, thereby maintaining virus persistence [128]. However, the characteristics and mechanisms of action of NEAT1 and MALAT1 in COVID-19 are still unclear.

Numerous studies based on bioassays have identified changes in NEAT1 and MALAT1 expression in COVID-19 that have been associated with viral replication [129,130]. For example, the change in MALAT1 expression found in patients with COVID-19 was previously considered to be a regulator of the expression of a gene encoding markers of lung cancer metastasis, and its deletion led to the activation of P53 and its target genes that affect the normal cell cycle [131]. However, recent studies have shown that MALAT1 can reduce epigenetic silencing of viral transcription by regulating promoter-enhancer interaction to promote viral transcription and infection [132]. The results of a number of other studies have shown that NEAT1 controls the processes of RNA regulation through the formation of nuclear paraspeckles of non-membrane organelles [133,134]. In addition, multiple studies have shown that MALAT1 and NEAT1 are expressed differently in patients with COVID-19 [129,130]. Studies on the role of MALAT1 in inflammatory lung injury in COVID-19 have shown that downregulation of MALAT1 facilitates inflammatory injury by inhibiting neutrophil chemotaxis and immune cell infiltration at the infected site [8,135]. Therefore, MALAT1 as a factor regulating neutrophil chemotaxis may be ubiquitous in severe cases, and its downregulation may play a role in alleviating inflammatory damage in patients with COVID-19 [8,135].

It was previously found that the immune response of monocytes and the replication of the COVID-19 virus require the support of aerobic glycolysis [136]. The transcription factor hypoxia-inducible factor 1-alpha (HIF-1a) can induce glycolysis, leading to a pro-inflammatory state in monocytes infected with COVID-19 [137]. It has also been previously found that HIF1A stabilizing long non-coding RNA (HISLA) in macrophages provides stability to HIF-1a by inhibiting its binding to the prolyl hydroxylase domain-containing protein 2 (PHD2) protein, thereby preventing its degradation and thus maintaining continuous activation of the aerobic state in tumor cells through HIF-1a signaling [138]. These data demonstrate the potential of lncRNAs as signaling agents that can be transmitted between healthy host cells and COVID-19 infected host cells via extracellular vesicles (EVs), promoting aerobic glycolysis in viral infections and tumors [139]. Accordingly, with respect to HIF-1a playing a similar role in COVID-19 infection, RNAi-mediated HISLA silencing may be a more appropriate method for inhibiting glycolysis in monocytes [140]. In addition, cell surface-attached nucleic acid aptamers have been reported to target the delivery of small interfering RNA (siRNA) to host immune cells, including macrophages. However, the strategy of using aptamer-miRNA-mediated HISLA knockdown, especially in associated macrophages, to treat COVID-19 needs further study. This chapter summarizes the involvement of lncRNAs in the regulation of COVID-19 (Table 4) [[141], [142], [143], [144], [145], [146], [147]].

**Table 4:** Summary of published studies on the role of long non-coding RNAs (lncRNAs) in COVID-19, with relevant literature highlighted.

LncRNA	Study design	Object	Gene-targets	Biological function	Reference
HOTAIRM1, MALAT1, PVT1 and AL392172.1	Bioinformatics	PBMC and BALF	Structural constituent of ribosome, chemokine activity, chemokine receptor binding, viral transcription, cytokine‐cytokine receptor interaction, IL‐17 signalling pathway and Nonsense‐Mediated Decay pathway	Promise to design the these lncRNA‐based drugs and to inspect the efficiency of vaccines	[141]
MALAT1 and NEAT1	Bioinformatics	NBHE	IFN responsive gene targets (IRF9, IFIT1, IFIT2, IFIT3, IFITM1, MX1, OAS2, OAS3, IFI44 and IFI44L)	Understanding the mechanism of COVID-19 progression with further exploration of lncRNAs data as potential biomarkers and therapeutic targets	[142]
MALAT1 and NEAT1	In silico, bioinformatics	A549 and Calu3 cells	IRF1, IRF4, STAT1, and STAT3	These lncRNAs might be involved in antiviral response	[143]
MEG3, MALAT1, NEAT1, DANCR, DGCR5, and DHRS4-AS1	Bioinformatics	hiPSC-CMs	IRFs, STAT1, STAT4, STA5A, STAT6, MYC, NFκB, and RelA/p65	These lncRNAs might be involved in COVID-19 pathogenesis	[144]
DANCR and NEAT1	Bioinformatics and in vitro	563 lung and blood tissues	Inflammation-related transcripts including IL-1β, IL-6, TNFα, NFkB1\2, Rel, and RelA\B	Associates with immediate and predicted long-term inflammation and neurological complications	[145]
NEAT1 and TUG1	In vitro	Human serum samples	CCL2, IL-6, and TNF-α	Involved in the development of cytokine storm	[146]
MALAT-1, NEAT-1, and THRIL	In vitro	PBMC	miR-155-5p	Could associated with the pathogenesis of COVID-19. Potential biomarkers for the monitoring and diagnosis	[147]

**Abbreviations:** HOTAIRM1, Hox antisense intergenic RNA myeloid 1; MALAT1, Metastasis-associated lung adenocarcinoma transcript 1; PVT1, Plasmacytoma variant translocation 1; NEAT1, Nuclear Enriched Abundant Transcript 1; TUG1, Taurine upregulated gene 1; DANCR, Differentiation antagonizing non-protein coding RNA; MEG3, Maternally expressed 3; DGCR5, DiGeorge syndrome critical region gene 5; DHRS4-AS1, DHRS4 antisense RNA 1; PBMC, Peripheral blood mononuclear cells; BALF, Bronchoalveolar lavage fluid; NBHE, Normal human bronchial epithelial cells; hiPSC-CMs, Human induced pluripotent stem cell-derived cardiomyocytes; IL‐17, Interleukin-17; IFN, Interferon; IRF9, Interferon regulatory factor 9; IFIT1, Interferon-induced protein with tetratricopeptide repeats 1; IFIT2, Interferon-induced protein with tetratricopeptide repeats 2; IFIT3, Interferon-induced protein with tetratricopeptide repeats 3; IFITM1, Interferon-induced transmembrane protein 1; MX1, Interferon-induced GTP-binding protein; OAS2, 2′-5′-oligoadenylate synthetase type 2; OAS3, 2′-5′-oligoadenylate synthetase type 3; IFI44, Interferon-induced protein 44; IFI44L, Interferon-induced protein 44 like; IRF1, Interferon regulatory factor 1; IRF4, Interferon regulatory factor 4; STAT1, Signal transducer and activator of transcription 1; STAT3, Signal transducer and activator of transcription 3; IRFs, IFN-regulatory proteins; STAT4, Signal transducer and activator of transcription 4; STAT6, Signal transducer and activator of transcription 6; NF-κB, Nuclear factor kappa-light-chain-enhancer of activated B cells; RelA, V-rel reticuloendotheliosis viral oncogene homolog A (avian); IL-1β, Interleukin-1β; IL-6, Interleukin-6; TNF-α, Tumor necrosis factor-alpha; NF-kB1\2, Nuclear factor kappa-light-chain-enhancer of activated B cells 1/2; RelA, V-rel reticuloendotheliosis viral oncogene homolog B (avian); CCL2, C–C Motif Chemokine Ligand 2.

### 7.3 MiRNAs, lncRNAs and antiviral therapy

The application of RNA interference technology using miRNA and lncRNA to inhibit COVID-19 replication in target cells is of great interest as a potential gene therapy for COVID-19 infection. In vitro miRNAs and lncRNAs against S protein, E protein, M protein, and N protein are able to limit their expression and suppress viral replication in human target cells. The most effective way to inhibit the replication of COVID-19 virus using RNA interference seems to be the impact on multiple regions of the virus genome or a combination of different therapy options [96,148].

The therapeutic antiviral effect of therapy may be achieved by direct action with the help of inhibitors and antagonists on miRNAs and lncRNAs of the host involved in the process of virus replication and spread. For example, during infection with the hepatitis C virus (HCV), miR-122 host miRNA in the liver selectively binds the 5′ end of the HCV genome, which contributes to the survival and accumulation of the virus in hepatocytes. MiR-122 specific inhibitor miravirsen (15-mer oligonucleotides) recognizes and blocks this miRNA, which leads to a decrease in viral load. Miravirsen was effective in vivo models and was the first miRNA antagonist included in clinical trials [96]. Based on the results of these studies, it was suggested that the targeted effect on miRNA, the regulation of which is impaired, can also be used to counteract COVID-19 infection.

The progression of ARDS to pneumonia is highly dependent on host genetic factors. Allelic variants of HLA class I have the most significant influence on the development of the disease [149]. The HLA-G locus has a significant association with the dynamics of COVID-19 infection [150]. Regarding the proposed link between HLA-G and COVID-19 infection, it has been suggested that during the early stages of inflammation, the host may produce the anti-inflammatory cytokine IL-10, which may subsequently upregulate HLA-G expression to avoid damage. These data were further complemented by a positive correlation next to interleukins in the acute phase of COVID-19 infection [151]. Thus, the expression and/or secretion of HLA-G may reflect a negative feedback response to inflammatory processes in viral infections. HLA-G expression is largely regulated by miR-148a and miR-152. Nucleotide substitutions in miR-148a and miR-152 cause disruption of its interaction with HLA-G mRNA, which leads to a high level of HLA-G expression [152]. These data became the basis for the development of new strategies for constructing miRNAs for the treatment of viral infections, including COVID-19.

Thus, miRNAs and lnRNAs appear to be promising drugs for therapy and diagnostics. Intensive research is underway to find appropriate inhibitors and ncRNA mimics for the treatment of various diseases, including viral infections.

## 8 Limitations of miRNAs-lncRNAs-based therapeutic and diagnostic approaches

Difficulties and challenges arise in the development of any drug or precise and specific diagnostic tool, and the design of therapeutic and diagnostic approaches based on miRNAs and lncRNAs is no exception. So far, there is insufficient knowledge about their function and mechanism of action in COVID-19. Nevertheless, this is a rapidly developing field of modern biomedicine/virology, and the number of candidate miRNAs and lncRNAs that are at the initial stages of clinical research demonstrates the great potential and promise of using miRNAs and lncRNAs for the diagnosis, prognosis, and therapy of a wide range of infectious diseases, including COVID-19.

In viral infections, including in the case of COVID-19 infection, the use of therapeutic approaches based on RNA interference is associated with a number of potential problems. With an inadequate choice, miRNAs and lncRNAs are able to increase viral activity to a greater extent than to inhibit it, as a result of increased formation of escape mutants during reverse transcription [103]. Therefore, it is extremely important to establish under what conditions the successful inhibitory action of miRNA and lncRNAs can lead to clinically significant results.

Risks include non-specific effects of miRNA and lncRNAs. For instance, miRNA molecules undergo modifications - uridinylation, adenylation, methylation - and the potential significance of these processes should be taken into account when designing regulatory miRNAs or their inhibitors [96]. It is also important to solve the problems of delivering therapeutic miRNA and lncRNAs to the appropriate target cells without side effects, including the development of technologies that could provide stable expression of miRNA and lncRNAs in vitro and in vivo. Extrapolation of the results of laboratory studies of the inhibition of viral replication in culture to the potential suppression of infection in the body requires caution and is one of the important problems in the development of miRNA/lncRNA drugs for the treatment of COVID-19 infection. Since miRNAs and lncRNAs have functional pleiotropy and cooperativity, it is necessary to investigate the possible cross-effects of other candidate miRNAs and lncRNAs as therapeutic drugs, as well as to determine the potential side effects of altering the regulation of miRNAs and lncRNAs and their reversibility. Once miRNA/lncRNA delivery and non-specificity problems are addressed in preclinical studies, they will not only become excellent targets for therapeutic agents, but will pave the way for a new generation of biomarkers in the diagnosis, prognosis, and monitoring of therapy for a wide range of human infectious diseases [96].

Regulatory processes mediated by viral miRNAs and lncRNAs are an important component of the interaction between the virus and the host. The identification of miRNAs and lncRNAs targets showed that viral miRNAs and lncRNAs play an important role not only in the life cycle of the COVID-19 virus, but also in determining the course of the disease, which is achieved by changing the activity of the host genome. This may be important for the escape of the virus from the action of the immune system by suppressing immune surveillance and extending the lifespan of host cells infected with COVID-19. The identification of such targets and the study of their functioning during COVID-19 infection can provide a clear picture of the interaction of the virus and the host and contribute to the development of innovative therapeutic strategies. miRNAs and lncRNAs are stable and represent an independent class of diagnostic and prognostic biomarkers. The involvement of these molecules in the escape of the virus from the action of the host immune response is a critical factor that requires in-depth analysis in the development of anti-COVID-19 vaccines and gene therapy approaches. The study of such important post-translational viral regulators has great diagnostic and therapeutic potential for COVID-19 infection [97,98].

## 9 SARS-CoV-2 - neurologic and cognitive impairments

SARS-CoV-2 arrives at specific neuronal and glial cell receptors directly through cerebrospinal fluid, olfactory and trigeminal nerves; neuronal dissemination and hematogenous pathways have also been described [10]. Hematogenous dissemination of COVID-19 or retrograde axonal transport during the early or later phase of infection leads to brain damage. Altered sense of smell or hyposmia in patients with COVID-19 requires exclusion of the possibility of CNS involvement. In the brain, the virus primarily affects the capillary endothelium, resulting in neuronal damage without marked inflammatory events. Subsequent rupture of cerebral capillaries and larger vessels can have severe consequences in patients with COVID-19. Experts suggest that SARS-CoV-2 not only affects the respiratory tract but also penetrates the CNS, causing neurological disorders. Mechanisms of possible involvement of the CNS are different [153]. It is possible that the development of respiratory failure accompanying a new coronavirus infection is related to the involvement in the pathological process not only of the lower airways, but also of the respiratory center in the brain stem. Epidemiological studies show that when coronavirus infection develops, the average time from the appearance of the first symptoms to the development of respiratory failure is 5 days. During this time, the virus can penetrate the blood-brain barrier (BBB) via the blood or transsynaptic route and affect brainstem neurons, thereby disrupting the respiratory center. Second, to invade cells, COVID-19 uses ACE2 as a receptor, which is found on the surface of neurons and glial cells in the brain. The interaction of the coronavirus with these receptors can result in direct damage to neurons without the development of inflammation. Third, special attention is paid to autoimmune mechanisms. The development of a cytokine storm in coronavirus infection increases the permeability of the BBB, making uncontrolled entry of viruses, bacteria, immune cells, toxic metabolites, and inflammatory agents into CNS structures possible.

Patients with any neurological manifestations tended to be younger than patients without them and showed a longer time from onset to hospitalization. In contrast, patients with encephalopathy were older than patients without it, had a shorter time from COVID-19 onset to admission, and had a greater history of any neurological disorder, cancer, heart failure, cerebrovascular disease, chronic kidney disease, diabetes, dyslipidemia, arterial hypertension, and smoking. More than two-thirds of patients with encephalopathy partially lose capacity after discharge and cannot serve themselves. In addition, among these patients there was the highest mortality rate: about 22%. The mortality rate among patients with other neurological symptoms does not exceed 3% [154].

The neurological complications of SARS-CoV-2-induced COVID-19 infection are manifold. They include an increased risk of stroke, encephalopathy, cerebral microbleeds, and autoimmune diseases such as acute polyradiculoneuritis (Guillain-Barré syndrome) [155,156]. Thus, neurological manifestations in the initial COVID-19 are noted in about 40% of patients, at hospitalization they are observed in more than half of patients and increase as the disease progresses, occurring in more than 80% of patients. The most frequent neurological manifestations are myalgias (44.8%), headaches (37.7%), encephalopathy (31.8%), dizziness (29.7%), dysgeusia (15.9%) and anosmia (11.4%). Generalized fatigue bothers half of patients at the debut of the disease and 80% during the disease [154]. Mental disorders such as depression, anxiety disorders, fatigue, and post-traumatic stress disorder have been identified following the acute stages of the epidemics preceding the coronavirus, but data on the effects of COVID-19 continue to accumulate [157]. In addition to the psychological stress associated with the pandemic, the direct effects of the virus itself and the subsequent host immune response on the human CNS and outcomes are still little known [158]. From various perspectives, viral infections are known to be epidemiologically common and some are harmful to the CNS due to the development of neuropsychiatric syndromes that affect the cognitive, affective, behavioral, and perceptual domains [159].

The study conducted by Guo et al. showed high rates of depression and anxiety among infected patients relative to controls [160]. It should be noted that psychiatric disorders may become more intense, as individuals with mild cognitive impairment have higher levels of apathy and anxiety, and patients with Alzheimer's disease also become more anxious and impaired motor activity, as well as comorbidities such as hypertension (60%), dyslipidemia (52.5%) and diabetes (30%), more often in women than in men [161].

It is also known that SARS-CoV-2 can be associated with neurological aspects and can affect various levels of the central and peripheral nervous system, in addition to skeletal-muscular damage, accounting for 36.4% of cases reviewed, including impaired consciousness such as ataxia, acute cerebrovascular disease, seizures and neuropathic pain [162].

The COVID-19 Mental Disorders Collaborators demonstrated that SARS-Cov2 pandemic resulted in a 27·6% increase in cases of major depressive disorders and 25·6% increase in cases of anxiety disorders globally [163].

The effect of COVID-19 on the CNS after successful treatment is not clear. There is an urgent need for clinical and laboratory studies to characterize the relationship between SARS-CoV-2 and neurologic complications and damage. The presence of a wide range of neurological complications associated with COVID19, such as ischemic or hemorrhagic stroke, encephalopathy and seizures suggests a direct effect of the virus on the CNS, an indirect effect through damage to other organ systems, or sporadic synergism between infectious mechanisms and comorbid conditions. A growing evidence suggests that the novel coronavirus is tropic to both vessels and nervous tissue. Larger and more systematic studies will be required to elucidate these pathogenic pathways.

Neurological complications associated with COVID-19 are quite common. These complications can put a person's life and functional abilities at risk. Early detection of post-covid neurological syndrome, control of risk factors, the process of rehabilitation after a coronavirus infection is an urgent and a very difficult task.

## 10 Conclusions

COVID-19 disease has a certain staging in the development of clinical manifestations, which are determined by the nature and severity of immunological disorders caused by the SARS-CoV-2 virus and the subsequent inflammatory response. An important role in the pathogenesis of the virus is played by the transmembrane protein ACE2, the expression of which in humans is genetically based and associated with a comorbid background, which makes it possible to identify high-risk groups in the population. Virus invasion into cells can occur via the CD147 spike protein. An important factor for infection and the development of a severe form of infection are genes associated with the signaling of inflammatory cytokines and chemokines, as well as mitochondrial genes involved in the regulation of homeostasis and inflammation.

Regulatory processes mediated by viral ncRNAs are an important component of the interaction between the virus and the host. The identification of ncRNA targets showed that viral miRNAs and lncRNAs play an important role not only in the life cycle of the virus, but also in determining the course of the disease, which is achieved by changing the activity of the host genome. This may be important for the escape of the virus from the action of the immune system by suppressing immune surveillance and increasing the lifespan of infected host cells. The identification of such targets and the study of their functioning during the course of the disease can provide a clear picture of the interaction of the virus and the host and contribute to the development of innovative therapeutic strategies. Determining the nature of disruption of miRNA-lncRNA-mediated regulation in COVID-19 infection will also allow identification of promising therapeutic targets. MiRNAs and lncRNAs are stable and represent an independent class of diagnostic and prognostic biomarkers. The involvement of these molecules in the escape of the virus from the action of the host immune response is a critical factor that requires in-depth analysis in the development of anti-COVID-19 vaccines and approaches to gene therapy. The study of such important post-translational viral regulators has great diagnostic and therapeutic potential for a wide range of diseases, including COVID-19 infection.

COVID-19 caused by the SARS-CoV-2 virus, along with damage to the respiratory system, can lead to the involvement of the central and peripheral nervous system. There is a relationship between severity. COVID-19 and the severity and frequency of neurological disorders. Severe neurological disorders predominantly complicate the severe course of COVID-19. Factors potentially complicating the course of COVID-19 and contributing to the development of neurological complications are hypertension, diabetes, heart disease, and chronic lung disease.

## Author contributions

Irina Gilyazova and Ilgiz Gareev: conceptualization, project administration and writing – original draft. Irina Gilyazova: validation. Ilgiz Gareev: writing – review and editing, investigation and resources. Yanina Timasheva and Alexandra Karunas: formal analysis and methodology. Anastasiya Kazantseva and Gulnaz Korytina: data curation. Albert Sufianov and Andrey Mashkin: visualization. Elza Khusnutdinova: Supervision and funding acquisition. All authors have read and agreed to the published version of the manuscript.

## Funding

This research was funded by the 10.13039/501100012190Ministry of Science and Higher Education of the Republic of Bashkortostan [agreement no. 1, 2 December 2022]; This work was supported by the Bashkir State Medical University Strategic Academic Leadership Program (PRIORITY-2030).

## Declaration of competing interest

The authors declare that the research was conducted in the absence of any commercial or financial relationships that could be construed as a potential conflict of interest.
